# Musculoskeletal pain in other body sites is associated with new-onset low back pain: a longitudinal study among survivors of the great East Japan earthquake

**DOI:** 10.1186/s12891-020-03234-0

**Published:** 2020-04-13

**Authors:** Yutaka Yabe, Yoshihiro Hagiwara, Takuya Sekiguchi, Yumi Sugawara, Masahiro Tsuchiya, Shinichirou Yoshida, Yasuhito Sogi, Toshihisa Yano, Takahiro Onoki, Tadahisa Takahashi, Jun Iwatsu, Ichiro Tsuji, Eiji Itoi

**Affiliations:** 1 Department of Orthopaedic Surgery, Tohoku University School of Medicine, 2-1 Seiryo-machi, Aoba-ku, Sendai, Miyagi 980-8574 Japan; 2 Division of Epidemiology, Department of Health Informatics and Public Health, Tohoku University Graduate School of Public Health, 2-1 Seiryo-machi, Aoba-ku, Sendai, Miyagi 980-8575 Japan; 3 Department of Nursing, Faculty of Health Science, Tohoku Fukushi University, 1-8-1 Kunimi, Aoba-ku, Sendai, Miyagi 981-8522 Japan

**Keywords:** Great East Japan Earthquake, Low back pain, Musculoskeletal pain, Natural disaster

## Abstract

**Background:**

Low back pain (LBP) is a common health problem experienced after natural disasters. LBP is often concurrent with other musculoskeletal pain; however, the effects of preexisting musculoskeletal pain on the development of LBP are not clear. The purpose of this study was to elucidate the association of musculoskeletal pain in other body sites with new-onset LBP among survivors of the Great East Japan Earthquake (GEJE).

**Methods:**

A longitudinal study was conducted with survivors of the GEJE. The survivors who did not have LBP at the 3 year time period after the GEJE were followed up 1 year later (*n* = 1782). Musculoskeletal pain, such as low back, hand and/or foot, knee, shoulder, and neck pain, were assessed with self-reported questionnaires. The outcome of interest was new-onset LBP, which was defined as LBP absent at 3 years but present at 4 years after the disaster. The main predictor was musculoskeletal pain in other body sites 3 years after the GEJE, which was categorized according to the number of pain sites (0, 1, ≥ 2). Multiple regression analyses were performed to calculate the odds ratio (OR) and 95% confidence interval (CI) for new-onset LBP due to musculoskeletal pain in other body sites.

**Results:**

The incidence of new-onset LBP was 14.1% (251/1782). Musculoskeletal pain in other body sites was significantly associated with new-onset LBP. Including people without other musculoskeletal pain as a reference, the adjusted OR and 95% CI for new-onset LBP were 1.73 (1.16–2.57) for people with one musculoskeletal pain site and 3.20 (2.01–5.09) for people with ≥ 2 sites (*p* <  0.001).

**Conclusions:**

Preexisting musculoskeletal pain in other body sites was associated with new-onset LBP among survivors in the recovery period after the GEJE.

## Background

Low back pain (LBP) is one of the most frequent health problems worldwide, and the point, 1-year, and lifetime prevalence of LBP range from 12 to 33%, 22–65%, and 51–84%, respectively [1, 2]. Moreover, LBP is among the leading causes of disability-adjusted life years [3, 4]; therefore, gaining an understanding of the factors related to LBP are important. Risk factors for LBP include age, sex, obesity, smoking, psychological distress, and sleep disturbance in the adult population [3, 5–8]. Further, musculoskeletal pain often occurs at multiple sites, and single-site pain has been shown to increase the risk of pain at other sites [9]. Indeed, some reports have found that LBP occurs concurrently with other musculoskeletal pain [10–12]. Most of these studies were cross-sectional; therefore, the association of preexisting musculoskeletal pain with new onset of LBP is not clear.

Musculoskeletal pain, including LBP, is reported to increase after natural disasters [13]. Disasters create severe stress in survivors due to fear of death, deprivation of sleep, the loss of housing and social relationships, all of which could lower pain threshold [13]. The Great East Japan Earthquake (GEJE), accompanied by a devastating tsunami, attacked the north-eastern coastal areas of Japan on March 11, 2011 [14]. This terrible disaster resulted in serious damage to these areas, and required a long period of reconstruction. A high prevalence of LBP has also been reported after the GEJE [15, 16], and previous longitudinal studies have revealed associated factors, such as subjective economic hardship and sleep disturbance [7, 16]. The prevalence of musculoskeletal pain apart from LBP (hereafter referred to as “other musculoskeletal pain”) was also increased in the recovery phase after the GEJE, and almost half of the survivors had musculoskeletal pain at multiple sites [17]. Since musculoskeletal pain could co-exist at multiple sites, we speculated that increased other musculoskeletal pain could be associated with new onset of LBP, and could lead to a high prevalence of LBP after natural disasters. Clarifying this association could be used to inform prevention and treatment strategies for LBP after a natural disaster. The aim of this study was to examine the association of other musculoskeletal pain with new-onset LBP in the recovery period after the GEJE longitudinally.

## Methods

### Participants

**Fig. 1:**
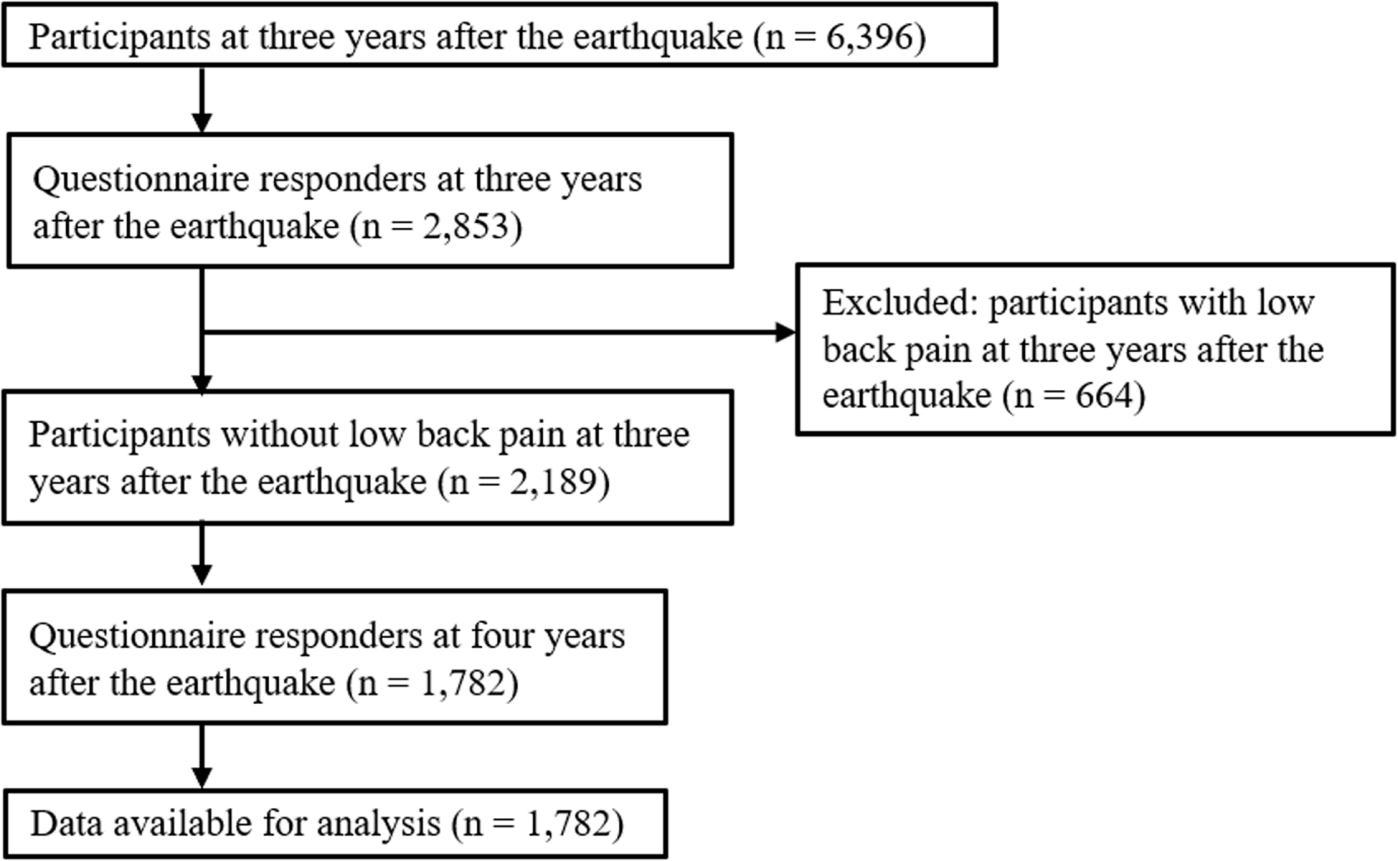
Flowchart of this study

We hypothesized that other musculoskeletal pain could be associated with new onset of LBP after natural disasters. A panel study was conducted with the GEJE survivors living in the severely damaged coastal areas, including Ogatsu and Oshika areas in Ishinomaki City, and Wakabayashi Ward in Sendai City, Miyagi prefecture, Japan [18]. The main purpose of the study was to understand the physical, mental, and social problems experienced by the survivors to provide effective support. The surveys began 3 months after the GEJE and were administered every 6 months. The first study population included residents registered in the Residential Registry of the Ogatsu and Oshika areas and survivors living in prefabricated housing in the Wakabayashi Ward [19]. Because the number of responders increased up to 3 years after the GEJE and remained constant after that period, we decided to examine the data at the 3 and 4 year time periods after the GEJE. From November 2013 to February 2014, 3 years after the GEJE, the residents (aged 18 years or over) who were registered in the Residential Registry of Ogatsu and Oshika areas, and the survivors who had participated in the previous survey in Wakabayashi Ward, were recruited (*n* = 6396). Self-reported questionnaires and informed consent forms were mailed to these residents and a 44.6% (2853/6396) response rate was obtained. Among those, the participants who already had LBP were excluded (*n* = 663). The remaining participants were followed from November 2014 to February 2015, 4 years after the GEJE, and an 81.4% (1782/2189) follow-up rate was obtained for this period. Finally, a total of 1782 participants were included in this study (Fig. 1). This study was approved by the institutional review board of our university (approval number: 201192) and was performed in accordance with the ethical standards as laid down in the 1964 Declaration of Helsinki and its later amendments or comparable ethical standards.

### Musculoskeletal pain

Musculoskeletal pain was assessed using self-reported questionnaires based on the Comprehensive Survey of Living Conditions. The questions were: “Have you had symptoms in the last few days? If yes, please place a mark next to all your symptoms.” The examples of choices were palpitation, dizziness, diarrhoea, and musculoskeletal symptoms such as low back, hand and/or foot, knee, shoulder, and neck pain [17]. The outcome of interest was new-onset LBP, which was defined as LBP absent at 3 years (first period), and present at 4 years (second period) after the GEJE. The main predictor was other musculoskeletal pain at the first period which included hand and/or foot, knee, shoulder, and neck pain. Other musculoskeletal pain was categorized into three groups according to the number of painful sites (0, 1, ≥ 2).

### Covariates

The following variables were included in the analysis because they were considered potential confounding factors in previous reports [16, 20]: sex, age, body mass index (BMI), living area, smoking habits, drinking habits, comorbid conditions (hypertension, diabetes mellitus, myocardial infarction, and cerebral stroke), working status, walking time per day, living status, subjective economic conditions, psychological distress (Kessler Psychological Distress Scale) [21], sleep disturbance (Athens Insomnia Scale) [22], and social isolation (Lubben Social Network Scale) [20].

### Statistical analysis

Univariate and multivariate logistic regression models were used to calculate odds ratios (OR) and 95% confidence intervals (95% CI) for new-onset LBP according to the number of other musculoskeletal pain sites in the first period. Variables included in the analysis were sex (male or female), age (continuous variable), BMI (continuous variable), living area (Ogatsu, Oshika, or Wakabayashi), smoking habits (non-smoker, smoker, or unknown), drinking habits (non-drinker, < 45.6 g of alcohol per day, ≥ 45.6 g of alcohol per day, or unknown), comorbid conditions (absence or presence of each comorbid condition), working status (unemployed, employed, or unknown), walking time per day (< 30 min, 30 min to < 1 h, ≥ 1 h, or unknown), living status (living in the same house as before the GEJE, prefabricated housing, new house, others, or unknown), subjective economic conditions (normal, a little bit hard, hard, very hard, or unknown), Kessler Psychological Distress Scale (continuous variable), Athens Insomnia Scale (continuous variable), and Lubben Social Network Scale (continuous variable). We further divided the participants into subgroups by sex (male or female), and ORs and 95% CIs for new-onset LBP were calculated in the same manner. For the stratified analysis, multiplicative interaction between other musculoskeletal pain and sex were tested using the Wald test. In addition, the ORs and 95% CIs for new-onset LBP according to each musculoskeletal pain except LBP in the first period were evaluated. We included the same variables (Model 1) and added each musculoskeletal pain such as hand and/or foot, knee, shoulder, and neck pain as covariates (Model 2). All statistical analyses were performed using SPSS 24.0 (SPSS Japan Inc., Tokyo, Japan). A *p* value of < 0.05 was accepted as statistically significant.

## Results

Baseline characteristics of the participants are presented in Table 1. Among the 1782 participants, 1343 (75.4%) had 0, 283 (15.9%) had one, 156 (8.8%) had two or more other musculoskeletal pain regions in the first period, respectively. The participants who reported having other musculoskeletal pain were more likely to be female and older. They were also more likely to have high BMI, comorbid conditions such as hypertension and myocardial infarction, short walking time, subjective economic hardship, higher scores on the Kessler Psychological Distress and Athens Insomnia Scales, and a lower score on the Lubben Social Network Scale (Table 1). The rate of new-onset LBP was 14.1% (251/1782). The crude and adjusted ORs and 95% CIs for new-onset LBP according to the number of other musculoskeletal pain regions are shown in Table 2. Other musculoskeletal pain was significantly associated with new-onset LBP in the crude and adjusted analyses. Including people without other musculoskeletal pain as a reference, adjusted ORs and 95% CIs for new-onset LBP were 1.73 (1.16–2.57) for people with one musculoskeletal pain site, and 3.20 (2.01–5.09) for people with ≥ 2 (p for trend < 0.001) (Table 2). The results for the stratified analysis are shown in Table 3. Other musculoskeletal pain was significantly associated with new-onset LBP in each group. The association was stronger in males compared with females (adjusted OR 3.16 (1.68–5.95) for people with one musculoskeletal pain site and 4.60 (2.06–10.30) for people with ≥ 2, p for trend: < 0.001 in males and 1.24 (0.71–2.14), 2.54 (1.38–4.68), 0.011 in females). There was no statistically significant multiplicative interaction between other musculoskeletal pain regions and sex (Table 3). For each musculoskeletal pain site, hand and/or foot, knee, shoulder, and neck pain were all associated with new-onset LBP in Model 1, and the association was also significant for knee and neck pain in Model 2. The adjusted ORs and 95% CIs (*p* value) for new-onset LBP were 2.04 (1.30–3.21, 0.002) for Model 1 and 1.33 (0.80–2.19, 0.27) for Model 2 where hand and/or foot pain were exposure variables; 2.56 (1.66–3.96, < 0.001) for Model 1 and 1.87 (1.16–3.01, 0.01) for Model 2 where knee pain was an exposure variable; 2.41 (1.32–4.39, 0.004) for Model 1 and 1.56 (0.82–2.95, 0.18) for Model 2 where shoulder pain was an exposure variable; and 2.61 (1.77–3.85, < 0.001) for Model 1 and 2.10 (1.40–3.17, < 0.001) for Model 2 where neck pain was an exposure variable, respectively (Table 4).

**Table 1:** Baseline characteristics of the participants

		Number of musculoskeletal pain sites except low back pain			
		0	1	≥ 2	*P* value
		1343	283	156	
Sex, n (%)	Male	678 (50.5%)	97 (34.3%)	51 (32.7%)	< 0.001
	Female	665 (49.5%)	186 (65.7%)	105 (67.3%)	
Age (18–97, years)**, mean (SD)	continuous variable	59.8 (18.3)	63.3 (16.1)	65.9 (13.8)	< 0.001
Body mass index (9.2–45.0)**, mean (SD)	continuous variable	23.9 (3.6)	23.9 (3.8)	24.8 (3.6)	< 0.001
Living area, n (%)	Ogatsu	573 (42.7%)	117 (41.3%)	80 (51.3%)	0.101
	Oshika	535 (39.8%)	112 (39.6%)	45 (28.8%)	
	Wakabayashi	235 (17.5%)	54 (19.1%)	31 (19.9%)	
Smoking habits*, n (%)	Non-smoker	1011 (75.3%)	204 (72.1%)	121 (77.6%)	0.154
	Smoker	263 (19.6%)	55 (19.4%)	24 (15.4%)	
Drinking habits*, n (%)	Non-drinker	794 (59.1%)	178 (62.9%)	101 (64.7%)	0.13
	< 45.6 g of alcohol/day	273 (20.3%)	54 (19.1%)	24 (15.4%)	
	≥ 45.6 g of alcohol/day	148 (11.0%)	20 (7.1%)	11 (7.1%)	
Comorbid conditions, n (%)	Hypertension	499 (37.2%)	123 (43.5%)	86 (55.1%)	< 0.001
	Diabetes mellitus	122 (9.1%)	28 (9.9%)	19 (12.2%)	0.444
	Myocardial infarction	70 (5.2%)	18 (6.4%)	20 (12.8%)	0.001
	Cerebral stroke	21 (1.6%)	8 (2.8%)	4 (2.6%)	0.282
Working status*, n (%)	Unemployed	696 (51.8%)	160 (56.5%)	95 (60.9%)	0.123
	Employed	619 (46.1%)	115 (40.6%)	59 (37.8%)	
Walking time/day*, n (%)	≥ 1 h	423 (31.5%)	69 (24.4%)	27 (17.3%)	< 0.001
	30 min to < 1 h	503 (37.5%)	92 (32.5%)	64 (41.0%)	
	< 30 min	394 (29.3%)	117 (41.3%)	62 (39.7%)	
Living status*, n (%)	Same house as before the GEJE	393 (29.3%)	90 (31.8%)	47 (30.1%)	0.494
	Prefabricated house	530 (39.5%)	103 (36.4%)	52 (33.3%)	
	New house	163 (12.1%)	34 (12.0%)	27 (17.3%)	
	Others	246 (18.3%)	55 (19.4%)	30 (19.2%)	
Economic condition*, n (%)	Normal	653 (48.6%)	114 (40.3%)	60 (38.5%)	< 0.001
	A little hard	347 (25.8%)	65 (23.0%)	43 (27.6%)	
	Hard	207 (15.4%)	77 (27.2%)	41 (26.3%)	
	Very hard	98 (7.3%)	26 (9.2%)	11 (7.1%)	
Kessler Psychological Distress Scale (0–24)**, mean (SD)	continuous variable	3.2 (4.0)	5.5 (5.0)	6.7 (5.0)	< 0.001
Athens Insomnia Scale (0–23)**, mean (SD)	continuous variable	3.3 (3.1)	5.3 (3.9)	6.2 (4.0)	< 0.001
Lubben Social Network Scale (0–30)**, mean (SD)	continuous variable	15.3 (6.0)	14.2 (5.6)	14.3 (6.2)	0.004

*Because each item has a limited number of respondents, the actual number is not necessarily in accordance with the total

**Indicates the range of the participants. GEJE; Great East Japan Earthquake, SD; standard deviation

**Table 2:** Association of musculoskeletal pain except LBP with new-onset LBP

	Number of musculoskeletal pain sites except LBP				
	total	0	1	≥ 2	P for trend
Participants	1782	1343	283	156	
New-onset LBP, n (%)	251 (14.1)	155 (11.5)	53 (18.7)	43 (27.6)	
Crude OR (95% CI)		1	1.77 (1.25–2.49)	2.92 (1.98–4.30)	< 0.001
Adjusted OR (95% CI)		1	1.73 (1.16–2.57)	3.20 (2.01–5.09)	< 0.001

Adjusted for sex, age, body mass index, living area, smoking habits, drinking habits, complications, working status, walking time, living status, subjective economic condition, Kessler Psychological Distress Scale, Athens Insomnia Scale, and Lubben Social Network Scale. LBP: low back pain, OR: Odds ratio, CI: Confidence interval

**Table 3:** Stratified analysis for each age group

	Number of musculoskeletal pain sites except LBP					
	total	0	1	≥ 2	P for trend	P-interaction
	1782	1343	283	156		
Sex						
Male (*n* = 826)						
New-onset LBP/participants	117/826 (14.2%)	75/678 (11.1%)	26/97 (26.8%)	16/51 (31.4%)		
Adjusted OR (95% CI)		1	3.16 (1.68–5.95)	4.60 (2.06–10.30)	< 0.001	
Female (*n* = 956)						0.12
New-onset LBP/participants	134/956 (14.0%)	80/665 (12.0%)	27/186 (14.5%)	27/105 (25.7%)		
Adjusted OR (95% CI)		1	1.24 (0.71–2.14)	2.54 (1.38–4.68)	0.011	

Adjusted for age, body mass index, living area, smoking habits, drinking habits, complications, working status, walking time, living status, subjective economic condition, Kessler Psychological Distress Scale, Athens Insomnia Scale, and Lubben Social Network Scale. LBP: low back pain, OR: Odds ratio, CI: Confidence interval

**Table 4:** Association of each musculoskeletal pain with new-onset LBP

		Absence	Presence	P value
Hand or foot pain	Participants	1616	166	
	New-onset LBP, n (%)	212 (13.1)	39 (23.5)	
	Model 1 OR (95% CI)	1	2.04 (1.30–3.21)	0.002
	Model 2 OR (95% CI)	1	1.33 (0.80–2.19)	0.27
Knee pain	Participants	1595	187	
	New-onset LBP	206 (12.9)	45 (24.1)	
	Model 1 OR (95% CI)	1	2.56 (1.66–3.96)	< 0.001
	Model 2 OR (95% CI)	1	1.87 (1.16–3.01)	0.01
Shoulder pain	Participants	1708	74	
	New-onset LBP	229 (13.4)	22 (29.7)	
	Model 1 OR (95% CI)	1	2.41 (1.32–4.39)	0.004
	Model 2 OR (95% CI)	1	1.56 (0.82–2.95)	0.18
Neck pain	Participants	1558	224	
	New-onset LBP	190 (12.2)	61 (27.2)	
	Model 1 OR (95% CI)	1	2.61 (1.77–3.85)	< 0.001
	Model 2 OR (95% CI)	1	2.10 (1.40–3.17)	< 0.001

Adjusted for sex, age, body mass index, living area, smoking habits, drinking habits, complications, working status, walking time, living status, subjective economic condition, Kessler Psychological Distress Scale, Athens Insomnia Scale, and Lubben Social Network Scale (Model 1). Additionally, adjusted for hand or foot pain, knee pain, shoulder pain, and neck pain (Model 2). LBP: low back pain, OR: Odds Ratio, CI: Confidence interval

## Discussion

The present study revealed that preexisting other musculoskeletal pain was associated with new-onset LBP among the survivors in the recovery period after the GEJE. Further, the effect was stronger with musculoskeletal pain that occurred at multiple sites.

Some cross-sectional studies have shown that musculoskeletal pain often occurs at multiple sites, such as shoulder, elbow, knee, and low back [23, 24]. Further, other authors reported a significant association between LBP and neck or knee pain [10–12]. A small number of longitudinal studies have investigated the effect of musculoskeletal symptoms on the onset of LBP. Smith et al. reported that preexisting pain resulting from arthritis or injury was associated with new-onset LBP [25]. Papageorgiou et al. showed that musculoskeletal pain history was a predictor of subsequent LBP [26]. The results of the present study reveal that the existence of musculoskeletal pain is associated with subsequent onset of LBP, which corresponds with these previously published reports. There has been speculation in the literature about the association between concurrent pain at different sites. Pain at one site can negatively affect motion or posture and place additional burden on the other parts of the body [27]. The factors associated with one pain can also be related to the other pain [28]. In addition, pain at one site can cause central sensitization which can result in the development of pain at other sites [11]. These conditions may explain the association between preexisting musculoskeletal pain and new-onset LBP.

To our knowledge, this is the first study to report that the effect of musculoskeletal pain on onset of LBP becomes stronger with multisite musculoskeletal pain, which may be considered a dose-response relationship. Nordstoga et al. reported that LBP with an increasing number of musculoskeletal pain sites tends to have a worse recovery rate, which also supports our results [29]. The association of musculoskeletal pain with LBP is stronger due to an increased number of pain sites. A high prevalence of musculoskeletal pain was reported after the GEJE, and many survivors experienced pain at multiple sites [17]. This is presumed to be one explanation for our finding of increased LBP after the GEJE. Attention should be paid to other musculoskeletal pain sites to treat and prevent LBP after natural disasters.

The stratified analysis according to sex categories revealed that the association of other musculoskeletal pain with new-onset LBP was also significant among categories in each group, which likely highlights the robustness of the association in this study. The rate of musculoskeletal pain was higher in females compared with males; however, the association of musculoskeletal pain with LBP was stronger in males. However, musculoskeletal pain, especially multisite pain, is more common among females [23, 24], and various factors, such as menopause and loss of oestrogen, may affect such pain [30], which is assumed to lower the association of musculoskeletal pain with LBP in females.

Some authors reported the association between LBP and hand or foot [31], knee [12, 28], shoulder [31], and neck pain [11] in cross-sectional studies. There have also been a small number of longitudinal studies regarding the association between LBP and each musculoskeletal pain, and preexisting LBP was reported to be associated with onset of knee [27] and neck pain [10]. To our knowledge, the present study was the first to report that preexisting knee and neck pain were individually associated with onset of LBP, even if the effect of the other musculoskeletal pain was considered. There is a closed kinetic relationship between the knee and lower back [12], and dysfunction of the knee joint due to pain may result in compensation and LBP. The spine undergoes a similar aging process, including genetic influences and risk factors to pain in the neck and lower back [10], which can cause LBP following neck pain. Some twin studies demonstrate that genetics are associated with the development of pain and that this association may further depend on age [32–34]. Further study is needed to clarify the mechanism of the relationships between LBP and neck pain. On the other hand, the association of hand and/or foot, and shoulder pain with LBP was not significant when considering the effect of the other musculoskeletal pain. Other musculoskeletal pain may also be associated with other pain, and that association may affect the results. Further, survivors who experienced LBP in the first period were excluded from this study, because the purpose of this study was to assess the effects of other musculoskeletal pain on LBP onset. The survivors who already had both LBP and other musculoskeletal pain were excluded, which could reduce the association.

The strength of this study includes a longitudinal design, large sample size, high follow-up rate (81.4%), and results that remained robust during stratified analyses. On the other hand, this study had several limitations. First, the questionnaires and informed consent forms were mailed to the participants, and the response rate for the first time-point was low at 44.6%. Although there is no information regarding non-responders, it might be that responders are healthier than non-responders, or that more severely affected persons may pay more attention to their situation, and may therefore be more likely to respond. These issues could affect the reported rate of musculoskeletal pain, and strengthen or weaken the association between other musculoskeletal pain and new-onset LBP. Further, this study examined the data at the 3 and 4 year time periods after the GEJE. The other time period had a different response rate, which could also affect the results. Second, musculoskeletal pain was assessed using a self-report questionnaire based on the Comprehensive Survey of Living Conditions. Although this survey is widely accepted in Japan as a tool to assess several participant characteristics (including symptoms), the reliability and validity of this method were not evaluated in this study. Further, the questionnaire included five pain sites but did not include other pain sites such as hip or elbow. Pain at these sites could also affect the onset of LBP and were not assessed in this study. In addition, pain severity and frequency were not assessed. The difference in severity and frequency of LBP might depend on the number of other musculoskeletal pain sites, which could not be assessed. Finally, this study was conducted with disaster survivors and generalizability of the results might be unclear. Future studies are required to determine if the findings of this study can be extrapolated to the general population.

## Conclusions

Preexisting musculoskeletal pain at other sites (especially knee and neck) was associated with new-onset LBP among survivors in the recovery period after the GEJE. Further research is needed to clarify whether this association is truly causal and to identify the mechanisms that could offer an explanation for why people with other musculoskeletal pain are at a higher risk of developing LBP. This could offer useful recommendations for clinicians and public health policies for LBP.

## Data Availability

The datasets used and/or analysed during the current study are available from the corresponding author on reasonable request.

## Abbreviations

BMI: Body mass index
GEJE: Great East Japan Earthquake
LBP: Low back pain
OR: Odds ratio
95%CI: 95% Confidence interval
